# Mode Coupling and Nonlinear Resonances of MEMS Arch Resonators for Bandpass Filters

**DOI:** 10.1038/srep41820

**Published:** 2017-01-30

**Authors:** Amal Z. Hajjaj, Md Abdullah Hafiz, Mohammad I. Younis

**Affiliations:** 1 Physical Science and Engineering Division, King Abdullah University of Science and Technology, Thuwal, 23955-6900, Saudi Arabia; 2 Department of Mechanical Engineering, State University of New York at Binghamton, Binghamton, NY 13902, USA

## Abstract

We experimentally demonstrate an exploitation of the nonlinear softening, hardening, and veering phenomena (near crossing), where the frequencies of two vibration modes get close to each other, to realize a bandpass filter of sharp roll off from the passband to the stopband. The concept is demonstrated based on an electrothermally tuned and electrostatically driven MEMS arch resonator operated in air. The in-plane resonator is fabricated from a silicon-on-insulator wafer with a deliberate curvature to form an arch shape. A DC current is applied through the resonator to induce heat and modulate its stiffness, and hence its resonance frequencies. We show that the first resonance frequency increases up to twice of the initial value while the third resonance frequency decreases until getting very close to the first resonance frequency. This leads to the phenomenon of veering, where both modes get coupled and exchange energy. We demonstrate that by driving both modes nonlinearly and electrostatically near the veering regime, such that the first and third modes exhibit softening and hardening behavior, respectively, sharp roll off from the passband to the stopband is achievable. We show a flat, wide, and tunable bandwidth and center frequency by controlling the electrothermal actuation voltage.

Microelectromechanical systems (MEMS) resonators have increasingly attracted the attention of researchers for applications^1^, such as filtering^2^, communications^3^, mass/gas sensing^4^,^5^, logic devices^6^, signal processing^7^, energy harvesting^8^, and sensors/actuators^9^,^10^.

Coupling of vibration modes has been a subject of increasing interest in recent years^11^,^12^,^13^,^14^,^15^. Those modes can be coupled mechanically (links) or electrically (external actuation). Also they can be coupled nonlinearly among the structure itself^12^ or through internal resonance^13^.

Thermal actuation has been used extensively to actuate bistable structures since it provides large displacement and a mechanism for resonance frequency tuning by low applied voltages^16^,^17^,^18^,^19^. In a recent work, it was shown theoretically and experimentally that large tunability can be achieved for a buckled beam by controlling the electrothermal voltage while exciting the resonator electrostatically^20^.

The intensive development of MEMS structures has led to a new generation of filters based on MEMS resonators thanks to the high-frequency selectivity and resonance frequency tunability^21^,^22^,^23^,^24^. To this end, electrically and/or mechanically coupled multiple MEMS/ Nanoelectromechanical systems NEMS resonators to realize bandpass filters has been the subject of intensive research^25^,^26^,^27^,^28^,^29^,^30^. Electrical coupling has an advantage over the mechanical coupling due to the ease of post-fabrication tuning of the filter characteristics. Hajhashemi *et al*.^27^ presented a tunable bandpass filter made of two electrostatically coupled MEMS resonators. They were able to tune the center frequency by controlling the DC voltage of the coupling electrode, and controlled the bandwidth by monitoring the applied axial stress. Other groups have investigated the ability to use two MEMS resonators that are tuned and excited independently and are electrically coupled to realize a bandpass filter. Lopez *et al*.^28^ presented a bandpass filter based on two clamped-clamped resonators with a resonance frequency around 22 MHz. They showed a bandwidth of 100–200 kHz in air, and 17 kHz in vacuum. Zou *et al*.^29^ investigated the coupling of four similar beams connected in a square ring to be used as tunable bandpass filter by controlling the electrostatic bias voltage. Yan *et al*.^30^ studied the ability to use four types of MEMS arrays for bandpass filter by internal mechanical and electrical phase inversion. Single MEMS resonator was used as notch or single-frequency pass filters with high quality factor in the vacuum condition^31^. Ouakad and Younis^32^ studied the dynamic response of an electrostatically excited arch beam resonator and proposed a filter operation based on the snap-through motion.

In this paper, we aim to realize a bandpass filter of sharp transition from the passband to the stopbands by utilizing the nonlinear jumps of two modes of vibration, which are coupled through the veering phenomenon. This phenomenon refers to the sudden veering of two eigenfrequencies as varying a control parameter instead of continuing their path where they cross. After the sudden veering, each frequency continues along the path that the other would have taken if they would to cross. The veering phenomenon has firstly reported by Liessa^33^ and then was investigated by several other studies^34^,^35^,^36^. It has been reported also for slacked CNTs when actuated by electrostatic forces^37^. To activate the veering phenomenon, we tune electrothermally the first and third resonance frequencies of a MEMS arch resonator so that they get very close to each other.

## Background

**Figure 1:**
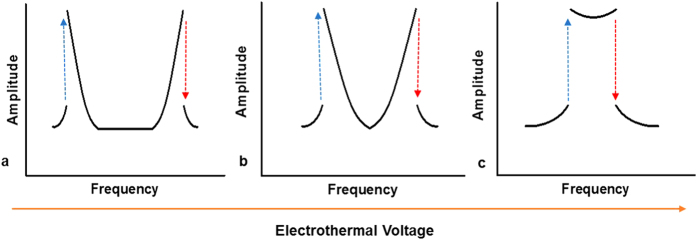
Schematic illustrating the proposed bandpass filter. (**a**) Shows the nonlinear response of the first (softening) and third (hardening) modes. (**b**) Shows the responses upon increasing the DC electrothermal voltage, thus bringing the two modes closer to each other. (**c**) Shows the two modes brought very close to each other (veering) and indicates the realization of the bandpass filter with jumps on the sides of the passband.

The arch beam under electrostatic force is subjected to a cubic nonlinearity, from mid-plane stretching, and a quadratic geometric nonlinearity, from curvature and electrostatic force, which can yield hardening and softening behavior of the various modes^38^. The nonlinear frequency response of the arch around the first and third (second symmetric) resonance frequencies is known to be dominated by the quadratic (softening behavior) and cubic (hardening behavior) nonlinearities, respectively. By changing a control parameter, voltage in this case, it is possible to bring both frequencies close to each other, veering, thus forming a band of frequency of high amplitude (the passband). This band will be sided by the two jumping frequencies, due to the softening and hardening behavior of the two modes. These jumps lead to sharp transition from the stopbands to the passband; thereby yielding near ideal bandpass filter. The concept is illustrated in Fig. 1.

## Materials and Methods

### Fabrication

**Figure 2:**
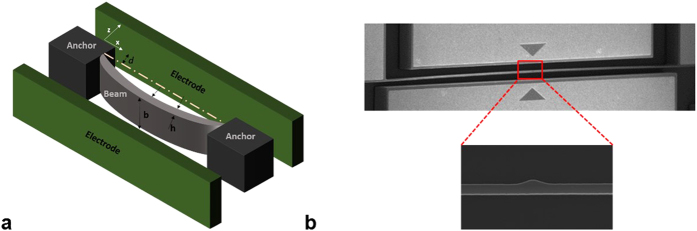
(**a**) A 3D schematic of the clamped-clamped arch beam. (**b**) Top view SEM picture of the actual device.

The resonator is fabricated on a highly conductive Si device layer of a silicon-on-insulator wafer by a two-mask process using standard photo-lithography, electron beam evaporation for metal layer deposition for actuating pad, deep reactive ion etch for silicon device layer etching and vapor hydrofluoric acid etch to remove the oxide layer underneath the resonating structure. The clamped–clamped arch beam, under consideration, is sandwiched between two adjacent electrodes, Fig. 2, to induce the vibration by exciting it electrostatically. The fabricated arch beam is of length 800 μm, width 30 μm, thickness 2 μm, and 2.6 μm initial rise.

### Experimental Setup

**Figure 3:**
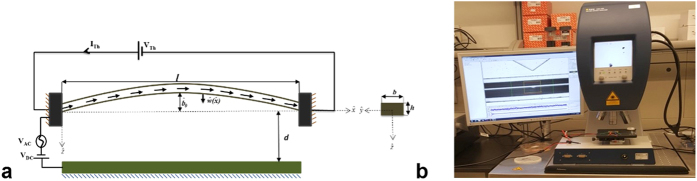
(**a**) Schematic of an electrothermally actuated clamped-clamped shallow arch. (**b**) Experimental setup.

The arch is actuated electrothermally by a DC voltage V_Th_ and electrostatically by a DC polarization voltage V_DC_ and an AC harmonic voltage of amplitude V_AC_, Fig. 3a. The electrothermal voltage V_Th_ is applied between the anchors of the arch inducing a current I_Th_ flowing through the microbeam that generates heat, which causes thermal expansion and controls its internally induced axial stress (compressive stress). This compressive force causes an increase in the microbeam curvature, and hence increases its stiffness.

Stroboscopic video microscopy from Polytec^39^, Fig. 3b, is used to determine the resonance frequencies as well as the frequency response of the arch beam. We measured the resonance frequencies of the arch beam using the ring down measurement and the fast Fourier transform (FFT) while varying the DC electrothermal voltage. To conduct frequency sweeps, we generate an amplified periodical sine signal to excite the in-plane arch. The stroboscopic video microscopy generates the frequency response curves in two different scales decibel and linear. It provides as well the frequency at −3 db. All the experiments are conducted in air and at room temperature.

## Results

**Figure 4:**
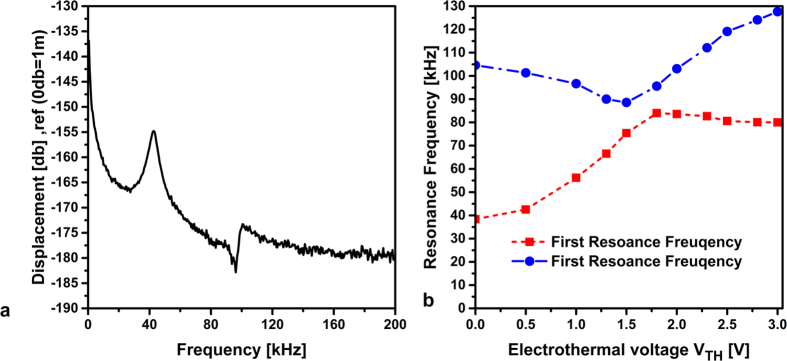
(**a**) FFT of the in-plane clamped-clamped arches at zero electrothermal voltage. (**b**) The variation of the first and third resonance frequencies of the shallow arch under electrothermal actuation.

Figure 4a shows the FFT of the arch beam under consideration for a zero electrothermal voltage. The first and third resonance frequencies of the unactuated arch beam are found to be around 38 kHz and 104 kHz, respectively.

One should note that the time associated with the electrothermal cooling and heating is much longer than the time associated to the vibration of the studied arch^40^. The associated thermal time coefficient could be calculated using the equation 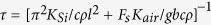. In the above equation *l, b* and *g* present the length, width of the arch beam and the gap between the arch beam and the substrate, respectively. *ρ, c, K*_*Si*_ and *K*_*air*_ are the silicon density, the silicon heat capacitance and the thermal conductivity of the silicone and the air, respectively. *F*_*s*_, the beam shape factor, is the correction term calculated based on the geometry of the arch beam using the formula^41^ given by 
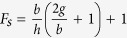
, where *h* is the thickness of the arch beam. In the studied case, *F*_*s*_is calculated to be equal to 17.995. Then the thermal time constant of the arch beam under consideration is 162.833 μs.

The variation in the measured resonance frequencies, while changing the electrothermal voltage V_Th_, is depicted in Fig. 4b. It can be observed that the first resonance frequency increases with the increase in the electrothermal voltage and reaches the value as high as twice of the initial value at zero electrothermal voltage. On the other hand, the third resonance frequency decreases while increasing the electrothermal voltage until getting very close to the first resonance frequency. At this critical electrothermal voltage, the third resonance frequency starts to increase while the first resonance frequency starts to become flat. Indeed, each frequency continues along the path that the other frequency would have taken. This phenomenon can be explained through the veering (avoided-crossing) phenomenon, which occurs when the resonance frequencies of two modes get close to each other^42^,^43^. Veering can be viewed as a way to mechanically couple the two involved modes.

**Figure 5:**
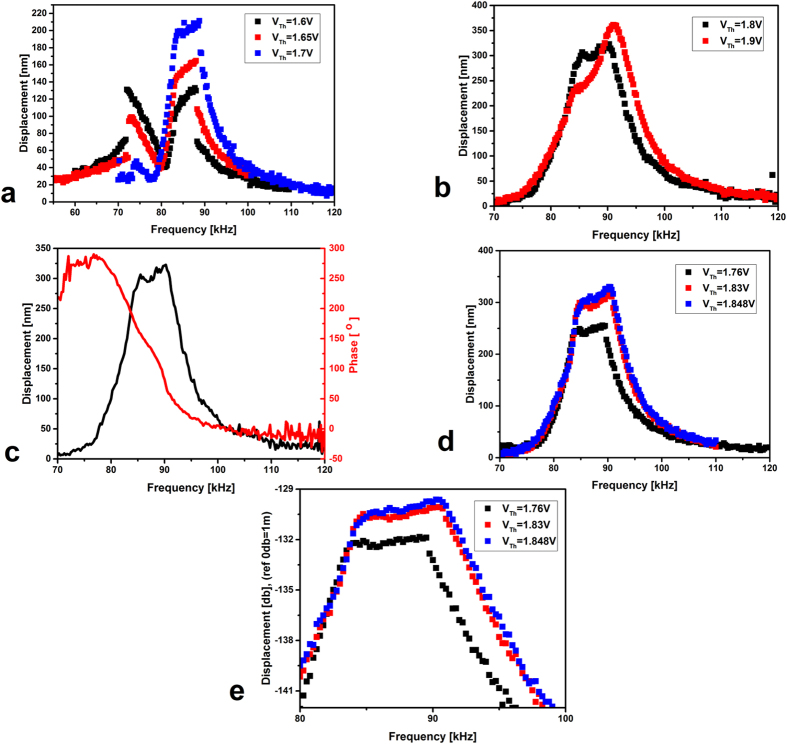
(**a**,**b**) Frequency response curves for V_DC_ = 20 V and V_AC_ = 20 V for different electrothermal voltages. (**c**) Frequency and phase response for V_DC_ = 20 V, V_AC_ = 20 V and V_Th_ = 1.8 V. (**d**) Frequency response for V_DC_ = 20 V and V_AC_ = 20 V at various electrothermal voltages. (**e**) Enlarged view of the bandpass in decibel scale.

Next, we excite the arch beam electrostatically by applying V_DC_ = 20 V and V_AC_ = 20 V for a range of electrothermal voltages where the first and third resonance frequencies are close to each other. For V_Th_ = 1.6 V, 1.65 V and 1.7 V, Fig. 5a, the first and third modes show softening and hardening nonlinear behavior, respectively, as expected of an arch microbeam^32^. This nonlinear behavior breaks the symmetry of the frequency response curve, which becomes no longer Lorentzian. However, for V_Th_ = 1.65 V, and 1.7 V, the third resonance frequency shows an amplitude of vibration more than the amplitude of vibration of the first resonance frequency and even more than the amplitude of vibration for both resonances for V_Th_ = 1.6 V. These results suggest that both modes start to interact with each other, where the third mode takes energy from the first mode, and hence eventually becomes of large amplitude.

For V_Th_ = 1.7 V, Fig. 5a, after a small softening jump, characterizing the first resonance frequency, a flat band of frequency starts to appear around the third resonance frequency. Upon increasing the electrothermal voltage to 1.8 V or 1.9 V, we are able to obtain a flat wide frequency band due to full interaction between the first and the third modes of vibration, Fig. 5b.

The flat band is a result of the combination of two resonance modes, the first and the third. We plot the experimentally obtained phase response for V_Th_ = 1.8 V, Fig. 5c. The total phase shift is shown to be around 300°, which suggests that there are two vibrational modes, the first and the third, getting close to each other, hence creating a flat passband around their corresponding resonance frequencies. This kind of phase response is typical for a bandpass filter^29^,^30^,^44^. Therefore, it is inferred that a flat bandpass filter can be realized based on electrothermally tuned single arch resonator by coupling the first and third mode of vibration.

Next, we characterize the tunability feature of this bandpass filter by varying the electrothermal voltage centered around 1.8 V, while keeping the electrostatic excitation force unchanged, Fig. 5d and e. Figure 5e is displayed in decibel scale to show the frequency at −3 db and then to extract accurately the center frequency and the bandwidth. It is shown that both the center frequency and the bandwidth can be moderately tuned by varying the electrothermal voltage.

Table 1 summarizes the bandpass filter features for different electrothermal voltages presented in Fig. 5d and e. We show a tunable center frequency f_0_ as well as tunable bandwidth Δf. The bandwidth, Δf, is wide, flat, and can be varied by 22% from 9 kHz at V_Th_ = 1.76 V to 11 kHz at V_Th_ = 1.848 V. The center frequency, f_0_, can be tuned by 3%.

**Figure 6:**
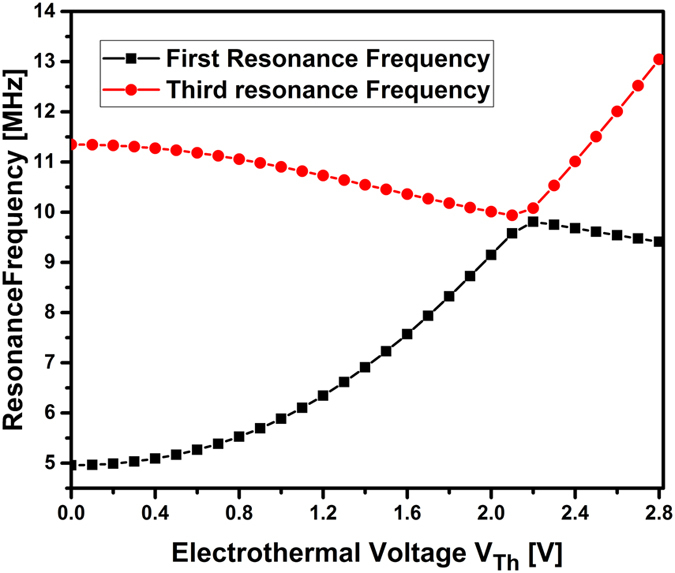
Variation of the first two symmetric resonance frequencies as tuning the electrothermal voltage for smaller arch of length 20 μm.

Note that these results can be improved for operation in the megahertz and gigahertz regimes by shrinking the dimensions of the arch beam and keeping the same concept of actuation. To demonstrate this, we conduct next theoretical simulations^21^,^33^ for a case study of smaller dimensions for an arch of length 20 μm, thickness 100 nm, width 850 nm, and initial rise 250 nm, Fig. 6. The figure shows the variation of the first two symmetric resonance frequencies as varying the electrothermal voltage; using the same thermal and mechanical parameters of the device under consideration in this paper. The figure indicates that as tuning the electrothermal voltage, that is equivalent to a compressive load, both frequencies get very close to each other with a separation near 80 kHz. This demonstrates that the same concept can be applied for smaller devices. One should note here that to enable the veering phenomenon, several parameters need to be carefully examined and chosen including the material properties, the nonlinearity from stress/strain, curvature, and the electrostatic force.

**Figure 7:**
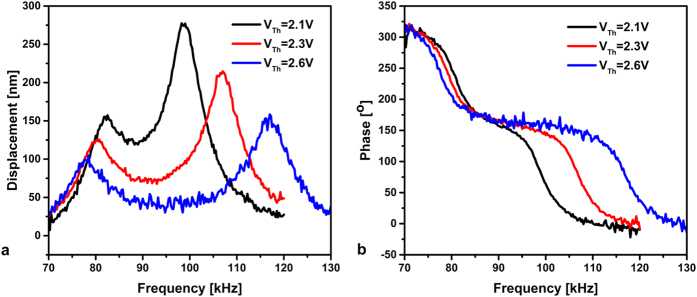
Frequency responses (**a**) and phase responses (**b**) for V_DC_ = 20 V, V_AC_ = 20 V, and various electrothermal voltages.

By increasing the electrothermal voltage for more than 2 V, Fig. 7, both resonance frequencies start to be further separated from each other. Figure 7 shows that the bandpass is destructed by the increase in the electrothermal voltage. However, the linear behavior is well-kept for the same excitation force.

## Conclusions

In this paper, we demonstrated experimentally a bandpass filter by exploiting the nonlinear hardening, softening, and veering phenomena in MEMS arches. The arches were electrothermally tuned and electrostatically actuated. We showed that the first resonance frequency of the MEMS arch beam increases with the increase in the electrothermal voltage until reaching a certain level after which it starts to saturate. Conversely, the third natural frequency decreases with the increase in the electrothermal voltage until getting close the first resonance frequency and after that it starts to increase. At some critical electrothermal voltages, the first and the third resonance frequencies get close to each other and get coupled by veering, which results in a bandpass filter with flat passband and wide bandwidth. In conclusion, we demonstrated that a single arch resonator could be potentially used as a tunable bandpass filter by a simple electrothermal frequency modulation scheme.

## Additional Information

**How to cite this article**: Hajjaj, A. Z. *et al*. Mode Coupling and Nonlinear Resonances of MEMS Arch Resonators for Bandpass Filters. *Sci. Rep.* **7**, 41820; doi: 10.1038/srep41820 (2017).

**Publisher's note:** Springer Nature remains neutral with regard to jurisdictional claims in published maps and institutional affiliations.

## Figures and Tables

**Table 1:** Tunability of the center frequency and bandwidth by the electrothermal voltage.

V_Th_ [V]	1.769	1.8322	1.848
f_0_ [kHz]	86.760	88.125	88.25
Δf [kHz]	9	10.25	11
